# Effectiveness of Health Impact Assessments: A Synthesis of Data From Five Impact Evaluation Reports

**DOI:** 10.5888/pcd13.150559

**Published:** 2016-06-30

**Authors:** Andrew L. Dannenberg

## Abstract

**Introduction:**

Since the 1990s, the use of health impact assessments (HIAs) has grown for considering the potential health impacts of proposed policies, plans, programs, and projects in various sectors. Evaluation of HIA impacts is needed for understanding the value of HIAs, improving the methods involved in HIAs, and potentially expanding their application. Impact evaluations examine whether HIAs affect decisions and lead to other effects.

**Methods:**

I reviewed HIA impact evaluations identified by literature review and professional networking. I abstracted and synthesized data on key findings, success factors, and challenges from 5 large evaluations conducted in the United States, Europe, Australia, and New Zealand and published from 2006 through 2015. These studies analyzed impacts of approximately 200 individual HIAs.

**Results:**

Major impacts of HIAs were directly influencing some decisions, improving collaboration among stakeholders, increasing awareness of health issues among decision makers, and giving community members a stronger voice in local decisions. Factors that contributed to successful HIAs included engaging stakeholders, timeliness, policy and systems support for conducting HIAs, having people with appropriate skills on the HIA team, obtaining the support of decision makers, and providing clearly articulated, feasible recommendations. Challenges that may have reduced HIA success were poor timeliness, underestimation of time and resources needed, difficulty in accessing relevant data, use of jargon in HIA reports, difficulty in involving decision makers in the HIA process, and absence of a requirement to conduct HIAs.

**Conclusion:**

HIAs can be useful to promote health and mitigate adverse impacts of decisions made outside of the health sector. Stakeholder interactions and community engagement may be as important as direct impacts of HIAs. Multiple factors are required for HIA success. Further work could strengthen the role of HIAs in promoting equity, examine HIA impacts in specific sectors, and document the role of HIAs in a “health in all policies” approach.

## Introduction

Health impact assessments (HIAs) have been used for 2 decades as a tool to facilitate communication between public health professionals and decision makers in other sectors (1). More than 390 HIAs in the United States have been completed or were in progress as of early 2016 (2). An HIA is a “a systematic process that uses an array of data sources and analytic methods and considers input from stakeholders to determine the potential effects of a proposed policy, plan, program, or project on the health of a population and the distribution of those effects within the population. HIA provides recommendations on monitoring and managing those effects” (3).

Several reports have called for evaluation to determine the impacts and usefulness of HIAs (3,4). Reasons to evaluate HIAs include assessing whether they provide the expected impacts, improving methods, identifying positive and negative unintended consequences, and justifying requests for future resources.

Three types of evaluation can be conducted on HIAs: process, impact, and outcome (3). *Process* evaluations examine the process followed in conducting an HIA and compares it with the practitioner’s intended plan or with established guidelines for HIA practice (5,6); several process evaluations have been published (7–10). *Impact* evaluations examine the impacts of an HIA on selected decisions and other events; such information is valuable to those who decide whether or not to conduct HIAs. *Outcome* evaluations examine changes in health status or health determinants caused by the HIA, but these are rarely conducted. The objective of this study was to review and describe 5 large impact evaluations of HIAs, 3 of which were recently published.

## Methods

I identified HIA impact evaluation reports by networking with HIA colleagues, searching literature in PubMed and Google Scholar, and reviewing references in published studies. I also searched major US and international HIA websites for relevant reports (2,11–15) and reviewed recent HIA textbooks (3,16–19).

This study included HIA evaluation reports available in English that assessed and synthesized data on the impacts of multiple individual HIAs. Of 8 potentially eligible reports identified, 1 unpublished student report (20), 1 unpublished conference presentation (21), and 1 Canadian study now in progress (22) were excluded. I abstracted information about background, methods, and findings, including the impact of HIAs on decisions and on other relevant events, factors that contributed to the success of HIAs, and factors that reduced the likelihood of an HIA having an impact. I reviewed the similarities and differences in impacts, success factors, and challenges found across the various reports. To compare multistudy HIA impact evaluations with individual HIA impact evaluations, I reviewed a convenience sample of 8 impact evaluation reports that described 11 individual HIAs (23–30) selected for topic and geographic diversity.

I invited the investigators of the 5 major HIA evaluation studies to review a draft of this report to ensure their studies were accurately described and to provide additional comments. Investigators who provided comments are named in the Acknowledgments.

## Results

### Characteristics of major HIA evaluation reports

Five reports synthesized the impacts of multiple HIAs (31–35) (Table 1). These reports covered HIAs conducted primarily in Europe, Australia, New Zealand, and the United States during various periods from 1996 to 2013. The number of HIAs reviewed per report ranged from 11 to 88 for a total of about 200 HIAs. Ten of the 23 HIAs reviewed by Bourcier et al (31) were among the 81 HIAs conducted in the United States and reviewed by Rhodus et al (34). A small but unknown number of HIAs conducted in Europe may have been reviewed by both Davenport et al (32) and Wismar et al (35).

**Table 1:** Characteristics of 5 Major Health Impact Assessment (HIA) Impact Evaluation Reports, United States, Europe, Australia and New Zealand, 2006–2015

Characteristic	Davenport et al, 2006 (32)	Wismar et al, 2007 (35)	Rhodus et al, 2013 (34)	Haigh et al, 2015 (33); Haigh et al, 2013 (36)	Bourcier et al, 2015 (31)
Organization	University of Birmingham, United Kingdom	European Observatory on Health Systems and Policies, Brussels, Belgium	US Environmental Protection Agency, Cincinnati, Ohio	University of New South Wales, Sydney, Australia	Group Health Research Institute, Seattle, Washington
Source of HIAs	Primarily Europe (85 of 88 HIAs [97%])	Europe: 19 countries	United States	Australia and New Zealand	United States
Years of HIAs reviewed	1996–2004	2002–2006	2005–2012	2005–2009	2005–2013
Sampling strategy	All HIAs found on multiple Web-based databases as of 2004	Purposely selected from list of 158 European HIAs completed or ongoing as of 2005 to “have some potential for effectiveness”	Used multiple databases of US HIAs completed as of spring 2012; chose all HIAs in 4 sectors related to agency mission	Purposely selected from among 55 Australia/New Zealand HIAs completed by 2009 to reflect willingness to participate and diversity in timing, geography, and effectiveness	Purposely selected from among all US HIAs completed as of 2013 for diversity in geography, sector, funding; subjectively successful
No. of HIAs reviewed	88	17 case studies, of which 8 were not HIAs as strictly defined	81	11	23
Level of decision making	Local or regional, 83; national, 4; supranational, 1	Local, 10; national, 6; multinational, 1	Local or county 63; state 13; national 4; unclear 1	Local or regional, 11	Local or regional, 17; state, 6
Sector	Transportation, 16; housing, 12; regeneration, 11; health care, 11; environment, 7; leisure, 7; industry, 5; other, 19	Transportation, 5; urban planning, 5; agriculture, 2; environment, 2; industry, 1; infrastructure, 1; nutrition, 1	Land use, 39; transportation, 21; housing/buildings/infrastructure, 17; waste management/site revitalization, 4	Land use, 7; health service, 2; housing, 1; transportation, 1	Built environment, 11; transportation, 3; natural resources/energy, 3; other, 6
Review methods	Reviewed 88 case studies and 32 HIA methods papers; conducted email survey of 10 academicians, practitioners, and policy makers	Worked with collaborators in each country to examine dimensions of effectiveness in case studies; included 3–6 interviews with stakeholders and decision makers for each HIA	Reviewed HIA reports; used minimum elements of HIA as defined by Bhatia et al (37) and 4-cell HIA effectiveness matrix (35); used Internet searches to assess impacts of HIA on decisions	Reviewed HIA reports and questionnaires completed by HIA practitioners; conducted 33 semistructured interviews with HIA stakeholders	Reviewed HIA reports; conducted 166 semistructured interviews with HIA team members, stakeholders, and decision makers; conducted Web survey of 144 HIA practitioners

None of the samples used in the 5 reports were representative of all HIAs. Davenport et al (32) reported on all HIAs identified in multiple databases as of 2004. The other reports purposefully selected HIAs from large databases by using criteria such as having “some potential for effectiveness” (35): reflecting diversity in sector, geography, timing, effectiveness, and funding (31,33): or relating to the agency’s mission (34). HIAs were examined from such sectors as land use, transportation, housing, agriculture, energy, health services, and waste management.

All 5 reports included a review of the written findings and recommendations for each individual HIA. Three reports (31,33,35) included interviews of key stakeholders and decision makers to assess impacts. Davenport et al (32) reviewed commentaries and discussion papers and included an email survey. Rhodus et al (34) used only publicly accessible documentation from the Internet to identify HIA impacts.

Each report sought to document whether HIA recommendations influenced the decisions they were intended to inform. All reports identified impacts on decisions of some HIAs and generally provided examples.

### Definition of HIA success

One reason to evaluate an HIA is to determine if it is a “success.” Davenport et al (32) reports that “a successful HIA is one where its findings are considered by decision makers to inform the development and implementation of a [policy, program or project].” Bourcier et al (31) states that “success for HIAs should therefore be defined by both their impacts on decisions and on the environments in which decisions are made.” Haigh et al (33) writes that effectiveness (success) can be defined as “the extent to which the HIA succeeds in bringing about the desired changes to decision-making and implementation . . . [but there are often disagreements] about what constitutes ‘success’ and what constitutes a ‘desired change.’” In an unpublished survey, HIA practitioners defined success as educating decision makers about the health consequences of a policy, influencing the design of a project, creating new partnerships between health and other agencies, facilitating community involvement in a decision, and addressing community concerns (21).

### Categories of effectiveness

Effectiveness categories could be helpful to distinguish useful from ineffective HIAs. Wismar et al (35) proposed 4 categories of effectiveness: *direct* (leads to changes in decision), *general* (raises awareness but no specific changes are made in decision), *opportunistic* (favorable decision would have been made anyway), and *ineffective* (HIA ignored in decision). Haigh et al (36) found these categories difficult to use because different aspects of a single HIA may fall into multiple categories, and judging whether an HIA impact fit into a category often needed a graded characterization rather than a yes-or-no decision.

Wismar et al (35) also proposed measuring effectiveness in 3 dimensions: *health effectiveness* (avoiding negative health effects and strengthening positive health effects), *equity effectiveness* (assessing and preventing disproportionate impacts on vulnerable populations), and *community effectiveness* (incorporating a community’s interests into the decision process). That review found varying levels of these dimensions of effectiveness across the 17 HIAs assessed. The other 4 reports did not use these dimensions in their analyses.

### Direct HIA impacts

The 5 reports I studied indicated that many HIAs directly influenced decisions (Table 2). Evidence fulfilling Wismar’s direct effectiveness criteria was found by Rhodus et al (34) in 30 of 81 HIAs (37%) and by Bourcier et al (31) in 11 of 23 HIAs (48%), although the 2 groups overlapped. Examples of decision changes directly attributable to an HIA include those associated with a county bicycle plan (26) and a housing proposal (38). A linear model of decision makers obtaining information, making a decision, and acting accordingly often does not reflect actual circumstances (33), and the impacts of many HIAs may not fit neatly into Wismar’s 4 categories. For some HIAs, other impacts such as community engagement may be more important than direct impacts.

**Table 2:** Findings and Impacts of 5 Major Health Impact Assessment (HIA) Impact Evaluation Reports, United States, Europe, Australia, and New Zealand, 2006–2015

Author and Year of Publication	Findings and Impacts
Davenport et al 2006 (32)	Important to monitor decisions to determine if impact occurred Engaging decision makers is important but may compromise independence and impartiality HIAs need to fit into the political and administrative environment in which they are being conducted; this fit may be as important as the technical methods used to conduct the HIA
Wismar et al, 2007 (35)	Described wide range of HIA methods used in 19 European countries Reported that some complex projects entail a large number of discrete decisions, so effectiveness may vary with different decisions Reported that none of the HIAs reviewed led to complete cancellation of a project **Frameworks** Defined 4-cell framework for overall effectiveness as direct, general, opportunistic, and none Identified dimensions of effectiveness as health effectiveness, equity effectiveness, and community effectiveness
Rhodus et al, 2013 (34)	Raised awareness of health and related issues Introduced health into discussions where health was typically absent (ie, informing decision making) Engaged community members and stakeholders in decisions that affect them Facilitated interdepartmental, interagency, and intersectoral collaborations Built relationships and capacity within the community For 50 of 81 HIAs for which impacts could be ascertained, effectiveness (28) was categorized as direct (60%), general (32%), opportunistic (2%), or none (6%) Only 13 of 81 HIAs (16%) met all the minimum elements as defined by Bhatia et al (37)
Haigh et al, 2015 (33); Haigh et al, 2013 (36)	91% of survey respondents reported that the HIA affected decision making 83% of those with HIA impacts reported that HIA recommendations were easily incorporated into planning process No respondent indicated that the HIA led to proposal postponement or cancellation Some HIAs influenced implementation of proposal after a decision was made Some HIAs helped legitimize involvement of the health sector in nonhealth sector decisions Many HIA participants reported technical, conceptual, and social learning from the HIA process **Frameworks** Findings generally supported Harris-Roxas and Harris (67) conceptual framework for HIA effectiveness The authors found Wismar’s 4-cell effectiveness framework (35) difficult to use **Concepts** Introduced concept of “proactive positioning” to recognize or create opportunities for conducting HIAs
Bourcier et al, 2015 (31)	48% of decision makers reported HIA shaped their decision making Made direct and concrete contributions from the recommendations to the decision-making process Facilitated incorporation of health objectives into plans, policies, and programs of nonhealth-related agencies Contributed to longer-term outcomes beyond initial decision targets Institutionalized or strengthened existing relationships between individuals and organizations, or created new and enduring relationships between public health and other agencies such as transportation or planning departments Helped decision makers and stakeholders see how health is connected to seemingly unconnected issues Built consensus around controversial topics Amplified community member voices in the decision-making process

The 5 reports indicated projects or policies are rarely cancelled because of unfavorable HIA recommendations. One HIA about rental housing voucher restrictions and another about a solid waste facility identified adverse health effects and contributed to those proposals not moving forward (34). Occasionally an HIA was favorable but the project was subsequently cancelled for other reasons (39).

### Other HIA impacts

Three reports highlighted the raised awareness of health issues among stakeholders and decision makers caused by the HIA (Table 2). Although difficult to quantify, this raised awareness has the potential to influence decisions, thus providing an impact that extends beyond the initial reason for which the HIA was conducted.

Two reports found that an HIA may help create and strengthen interactions among stakeholders and other community members. Such interactions may continue after the HIA is completed and provide ongoing community benefits. Many HIAs helped facilitate the relationships between agencies across sectors (40).

Two reports emphasized the value of community engagement as part of conducting HIAs. The HIA process is designed to incorporate community input and may amplify community member voices in the decision-making process (31,41). Through participation in HIAs, community members may take action, increase contact with decision makers, and acquire skills that could help them influence decisions (42). Community engagement with the goal of empowerment is central to the HIA core value of promoting equity (43,44).

Other potential HIA impacts mentioned in various reports include developing new cross-disciplinary and interagency collaborations, identifying data gaps and questions for future research, establishing a foundation for appropriate monitoring, ensuring the public has accurate information on adverse and beneficial effects, and developing new forecasting methods (3).

### Success factors

The 5 reports identified factors that contribute to success (also called “enablers” [32]) (Table 3). The following factors were identified in at least 2 reports. The wording of success factors and of challenges varied widely among reports.

**Table 3:** Success Factors in 5 Major Health Impact Assessment (HIA) Impact Evaluation Reports, United States, Europe, Australia, and New Zealand, 2006–2015

Author and Year of Publication	Success Factors
Davenport et al 2006 (32)	**Role of decision makers** Involvement of decision makers/key stakeholders in the planning and conduct of the HIA (for example, commissioning, steering group, formulation of recommendations) Input from professionals outside of the usual range of people involved in the decision-making process Balance between decision maker ownership and HIA credibility **Policy making process and environment** Clear commitment to HIA within organizational decision-making structure Not being a controversial issue Policy support for HIAs (including supporting legislation, promotion of consistency of methods, monitoring, and evaluation) Provision of an enabling structure for HIA (manpower, evidence base, and intersectoral working) Existing statutory frameworks supporting the use of HIAs Recommendations chime with other political drivers Recommendations realistic and can be incorporated into the existing planning process **Timing of HIAs** Timing of assessment should fit with the decision-making process HIAs need to fit with decision makers’ rules, procedures, and time frames **HIA methods** Use of a consistent methodological approach Consideration of a broad range of factors that can have an impact on community health and safety Inclusion of empirical evidence relating the effects of a policy, program, or project on health Quantification of impacts Conduct by expert assessors (credibility of results) **Methods of reporting HIAs** Tailored presentation of information Use insight into organizational concerns and priorities to shape recommendations
Wismar et al, 2007 (35)	Capacity to deal with community pressure Timing in relation to the decision-making process Involvement of organizations that can support conduct of the HIA Culture of public health in the country Political leadership Public support Involvement in early stage of proposal development Legal backup for using health determinants in assessment Creation of health systems units to support HIA Clarification of who bears costs of HIA
Rhodus et al, 2013 (34)	**HIA best practices listed as** Adherence to minimum elements of HIA as defined by Bhatia et al (37) or to National Research Council (3) criteria Use of HIA as a tool for environmental impact assessment Promotion of health equity Documentation of screening and scoping Rules of engagement memos Communication plans Stakeholder involvement Transparent documentation of literature searches/reviews Use of best available qualitative and quantitative data Evaluation of quality of evidence Identification of data gaps Use of existing tools, metrics, methods, and standards Adaptation of existing tools and methods Detailed documentation of data and methods Use of geographic information systems Use of impact pathways and logic frameworks Clear summary of impact assessments Confidence estimates and assessments of uncertainty Documentation of process for prioritizing recommendations Recommendations that meet established feasibility criteria Development of an implementation plan for recommendations Clear and transparent HIA reporting Process evaluation Establishment of monitoring plans for impact and outcome evaluation
Haigh et al, 2015 (33); Haigh et al, 2013 (36)	Use of a structured stepwise process Flexibility to adapt process to local context Use of evidence to support recommendations Capacity and experience among practitioners and stakeholders Involvement of decision makers and others who can influence decisions or implement recommendations High-quality relationships across sectors Engagement of community stakeholders Shared goals and values among HIA participants Use of “proactive positioning” to achieve optimal timing Flexibility in time and timeliness to conduct HIA
Bourcier et al, 2015 (31)	Method of screening and choosing HIA targets wisely because an HIA is not always the right tool Investment in the right team to conduct HIA Engagement of key stakeholders Engagement of decision makers throughout the process Development of clearly articulated recommendations that spark action Delivery of compelling messages to the right audiences at the right times Use of approach to take advantage of HIA credibility

- **Engaging stakeholders.** All 5 reports suggested that stakeholder input was valuable during the planning and conducting of the HIA, generally to provide a unique perspective and to increase knowledge and skills among the HIA team and the decision makers.
- **Timeliness.** Four reports suggested that an HIA must be conducted early enough in the decision process to have an impact.
- **Policy and systems support for conducting HIA.** Three reports indicated the value of having frameworks or legislation supporting HIA conduct and of following established guidelines for conducting HIAs.
- **Engaging the “right” people on the HIA team.** Three reports found that team members may be chosen based on their knowledge and experience with HIAs, teamwork skills, knowledge of the subject matter being assessed, and acquaintance with the community involved.
- **Engaging and obtaining support of decision makers.** Three reports highlighted the importance of ensuring the willingness of decision makers to receive and consider HIA recommendations.
- **Clearly articulated, feasible recommendations.** Three reports highlighted the value of clear, precise language to make it practical for decision makers to act on the HIA recommendations.
- **Tailoring presentation of messages to decision makers and other audiences.** Three reports indicated that HIAs with summaries of findings and recommendations written in language that is appropriate for the intended audiences are more likely to achieve the desired impact.

Other success factors included taking advantage of HIA credibility to influence decision makers, quantifying health impacts, clarifying who pays the HIA costs, and using proactive positioning to recognize or create opportunities for HIAs and optimize HIA timing. These factors were each reported in one evaluation. One study reported development of an implementation and monitoring plan for recommendations as a success factor, but another study reported inadequate follow-up of recommendations as a challenge.

### Challenges

The reports identified various challenges to an HIA being considered successful (Table 4). The following challenges were identified in at least 2 reports.

**Table 4:** Challenges in 5 Major Health Impact Assessment (HIA) Impact Evaluation Reports, United States, Europe, Australia, and New Zealand, 2006–2015

Author and Year of Publication	Challenges
Davenport et al, 2006 (32)	**Role of decision makers** Limited organizational unique HIA conducted by champions external to the decision-making organization Not having the support of decision makers **Policy-making process and environment** Lack of awareness of health issues by nonhealth-related sectors Lack of knowledge (on behalf of those conducting HIA) of the policy-making environment HIA not a statutory or policy requirement **Conduct and reporting of HIAs** Lack of an established standard method for conducting an HIA Time, resources, and staffing Use of jargon
Wismar et al, 2007 (35)	HIA timing Quality of communication among stakeholders Quality of HIA predictions Conflicting objectives between health and other sectors in which HIA is done Links among local, national, and international decision making Lack of institutionalization of HIAs Uneven development of HIAs across countries
Rhodus et al, 2013 (34)	Ability to discern impact of HIAs on decision-making processes by Internet searches is limited **Areas for improvement** Increase adherence to the minimum elements of HIA as defined by Bhatia et al (37) or to National Research Council (3) criteria Expand use of HIA to inform decision making at local, state, and national levels Use consistency in HIA terminology Expand use of existing tools and resources for HIAs Identify and close data gaps
Haigh et al, 2015 (33); Haigh et al, 2013 (36)	Dealing with problem makers and proposal opponents Responding to unanticipated events such as change in decision maker Identifying effectiveness when goals of HIA were not explicit
Bourcier et al, 2015 (31)	Underestimation of overall level of effort Engagement of stakeholders and decision makers Pace of decision making and political administration changes Lack of access to relevant data Incorporation of equity and vulnerable populations consistently and meaningfully Follow-up on recommendations

- **Timing and timeliness.** Three reports indicated that HIAs may have little impact if completed too late to affect a decision. For example, in legislative settings, the pace of decision making is sometimes faster than the pace of the HIA.
- **Adequacy of resources.** Three reports found that practitioners often underestimated the time and staff resources needed to conduct an HIA.
- **Accessing relevant data.** Two reports found that the absence of sufficiently local health data may reduce the value to decision makers of an HIA’s findings and recommendations.
- **Use of jargon.** Two reports mentioned that use of technical terms or inconsistent terminology in HIA reports may reduce their value.
- **Lack of support from, or involvement by, decision makers**. Two reports found that HIAs are unlikely to affect decisions when decision makers are uninvolved in the process.
- **Absence of requirement to consider HIA findings.** Two reports mentioned that decision makers may give minimal consideration to recommendations by an HIA that was not required.

Other reported challenges included being involved in controversial topics, lack of knowledge of HIA practitioners about policy and of policy makers about health, and disagreements on the breadth of the definition of health and on expert predictions in HIA reports.

### HIA evaluation of individual HIAs

A review of impact evaluations of a convenience sample of 11 individual HIAs in 8 reports (Table 5) found impacts, success factors, and challenges similar to those described above. These impact evaluations were done by investigators associated with conducting the original HIAs, except for the 4 HIAs in Ireland (29). A New Zealand website has a list of 13 individual HIA impact evaluations (45), but it does not synthesize the common findings across those evaluations. No similar lists were identified for individual HIA evaluations conducted elsewhere.

**Table 5:** Impact Evaluations of Selected Individual Health Impact Assessments (HIAs) in Various Countries, 2004–2013

Author and Year of Publication	Name and Location of HIA	Sector	Example of HIA Recommendation Adopted in Final Project or Plan	Selected Feature, Finding, or Impact
Mindell et al, 2004 (23)	London’s draft transport strategy HIA, United Kingdom	Transportation	Giving priority to infrastructure and services that benefit London’s economically deprived communities	Achieved policy changes and raised awareness of health issues among policy makers
Mathias and Harris-Roxas, 2009 (24)	Christchurch Urban Development Strategy HIA, New Zealand	Land use	17 actions (not specified) in final strategy addressed HIA recommendations	Included process and impact evaluation; strong cross-sector relationships contributed to HIA success
Quigley and Watts, Ltd, 2010 (25)	Evaluation of the Whānau Ora HIA of the Draft Wairarapa Alcohol Strategy, New Zealand	Alcohol policy	HIA recommendations led to revisions of draft strategy	Public health team conducting HIA increased its knowledge and skills with HIA
Clark County, 2011 (26)	Clark County Bicycle and Pedestrian Master Plan HIA, Clark County, Washington	Transportation	Health and equity incorporated as project selection criteria	Of 11 HIA recommendations, 8 were fully adopted and 3 partially adopted
Harris-Roxas et al, 2011 (27)	Equity-focused HIA on health promotion policy implementation plan, New South Wales, Australia	Health promotion policy	Change in resource allocation split between rural and urban services	Focused on equity aspects of a health-sector plan
Ross et al, 2012 (28)	Atlanta BeltLine transit, parks, and redevelopment project HIA, Atlanta, Georgia	Land use and transportation	Public health professional added to project advisory board	Explicitly tied findings to recommendations and to subsequent impacts
O’Mullane and Quinlivan, 2012 (29)	Four policy HIAs on transport, housing, community development, air quality plan, Ireland	Multiple sectors	Transport HIA “was used to plan further health promotion and community planning activities”	Found local government can be an enabler or barrier to success of HIA
Canterbury District Health Board, 2013 (30)	Evaluation of the HIA of the Canterbury Regional Land Transport Strategy, New Zealand	Transportation	Committing future funding to policies supporting active transport and public transport	Noted that HIA report recommendations did not include effective monitoring for health issues

## Discussion

The evaluation reports reviewed consistently showed that some HIAs had direct impacts on decisions. Others may have had no direct impact on a decision because the decision maker was not receptive or the decision process was complex and nonlinear. Other impacts were found for most HIAs and may have been as important as direct impacts. Such other impacts included raising awareness of health issues among decision makers, building new partnerships among stakeholders, and engaging community members in issues that affect them. HIAs rarely led to project cancellations, which may be a concern especially for projects in the private sector.

Various success factors and challenges were identified across the studies, but few were consistently found in all reports. No single success factor was identified as necessary and sufficient to ensure the success of an HIA. Even timeliness was found to be not essential; Haigh et al (33) reported examples in which the HIA affected not the decision but the subsequent implementation of the policy.

The findings on HIA success factors and barriers of this report are consistent with the findings of other studies. One survey of 47 HIAs in the United States documented the importance of community participation to increase the likelihood of HIA success (42). A British study based on 14 key informant interviews reported that leadership, integration of the HIA with existing organizational structures, and collaboration among key stakeholder organizations were central to encouraging HIA use in decision making (46). A master’s thesis that reviewed 54 HIAs in the United States reported that time and funding constraints are often barriers to HIA effectiveness and that most HIA practitioners believe that the use of quantitative health data strengthens HIA credibility (20).

A survey of participants in the European Healthy Cities Network reported that the following factors facilitated the use of an HIA: political support, training in HIA, collaboration with a public health institution or agency, a culture of intersectoral collaboration, a supportive national policy, and access to HIA expertise (47). The same survey reported the following barriers to HIA implementation: lack of skill, knowledge, and experience about HIAs; lack of a legal basis for implementation; and lack of political support (47). In a related report, factors enhancing the acceptability of recommended interventions included documented benefits, adaptability for stakeholder needs, an evidence base for the work, engagement of stakeholders, and the use of simple, clear language (48).

Promoting equity and reducing health disparities are often cited as major reasons for conducting an HIA (43,44,49). Equity may have improved as a result of some HIAs examined in this study, but no reports explicitly documented changes in equity or the factors contributing to reduced inequities. This lack of documentation may be due to the time required for documentation or difficulty in measuring such changes.

Process and outcome evaluations are complementary to impact evaluations. Results of several process evaluations of HIAs have been published, either alone (7–10) or as a combined process and impact evaluation (34,35). Guidelines are available for process evaluations (17,50). Most process evaluations have found substantial variability in the extent to which HIA investigators followed guidelines for HIA practice (8,34).

HIA outcome evaluations would be valuable to document whether implementing a recommendation has actual effects on health outcomes (eg, rate of heart disease) or on health determinants (eg, physical activity level) (3,51). Outcome evaluations are difficult to conduct because 1) causal pathways are complex, making it difficult to attribute a specific change in a health outcome or determinant to a specific HIA recommendation, 2) there may be no comparison group to document what would have happened in the absence of the HIA, and 3) they require substantial time and resources (3,19,51,52). No outcome evaluations were found in my literature review. The most relevant example found was a class exercise that predicted dietary changes related to a new supermarket (53).

This analysis synthesized data on the characteristics and findings of multiple HIA impact evaluations. It found compatible results across 5 major reports that evaluated more than 200 individual HIAs. These reports examined HIAs that were conducted in diverse sectors, countries, and years, and by various methods. Particularly valuable were the interviews of stakeholders and decision makers in 3 reports, an approach that increases the likelihood of identifying which factors influence a decision. This analysis also compared various success factors and challenges that affect HIA impacts across multiple studies.

This study has several limitations. The contexts and health systems vary across the countries in which the HIAs were conducted. HIA evaluations may have been missed if unpublished or if published in a language other than English. HIAs conducted in countries whose primary language is not English, such as Iran (54), Mongolia (55), and Thailand (56), may have different impacts on decisions, because of cultural, political, or economic differences. Each of the 5 evaluations used a different strategy for selecting HIAs; none were random samples of all HIAs. Three evaluations included potential effectiveness as part of the sampling strategy, which may have biased results toward finding an HIA to be effective. For some HIAs, decisions were being made as the HIA was being evaluated, so subsequent impacts of the HIA may have been missed. For other HIAs, the decision maker may not have remembered the impact of the HIA on a decision made long ago. Some decision makers may not have been able to state exactly how much weight was given to each of several factors (eg, health, economic, political, and social) in reaching a decision. Some HIAs involved politically sensitive topics about which informants may have been reluctant to provide full information. Some findings were difficult to quantify, such as the extent to which collaboration increased and awareness of health issues among stakeholders was raised. Not all of the evaluators interviewed decision makers; information obtained from HIA investigators or public sources may have not included data on influences of the HIA on the decision-making process. Finally, little information was available about the methods or impacts of HIAs conducted in the private sector (3).

The findings of this review lead to additional questions for which further research would be valuable. Several of these questions are now being researched by working groups of the Society of Practitioners of Health Impact Assessment (57).

First, because the impacts of HIAs are complex, no simple answer exists for policymakers and funders who ask how HIAs make a difference in decision making. HIAs may be more effective in some sectors than in others. Guidelines for best practices for HIAs in various sectors could be developed from reviews and case studies of HIAs in each sector (39,58,59; L.N. Gase et al, unpublished data, 2016).

Second, further work is needed to document the value of HIA in promoting equity and reducing health inequities. Such work requires identifying appropriate indicators of equity that are likely to be affected by decisions and for which changes can be measured (60). 

Third, outcome evaluations would be valuable to document the usefulness of HIAs. It may be possible to design outcome evaluations of some HIAs that focus on changes in selected health determinants, such as physical activity or diet. 

Fourth, HIAs are a valuable tool in a health-in-all-policies approach to incorporating health into policies in many sectors (61–63). Further work could document examples in which HIAs were used to advance this approach. Impact and outcome evaluations in settings in which health-in-all-policies approaches were implemented could be conducted.

Fifth, HIAs range from rapid desktop analyses to comprehensive studies that require substantial time and resources. Further work could lead to guidelines on the depth and breadth of HIAs needed for projects and policies in various contexts. For some HIAs, such as those for living wage policies in multiple cities (64–66), the findings and recommendations may be generalizable, thereby making further HIAs on the topic unnecessary. 

Sixth, the importance of an implementation and monitoring plan in the recommendations of an HIA is often underemphasized by HIA practitioners. Further work could establish best practices to routinely create such plans in HIAs and facilitate follow-up of these plans. 

Finally, some HIAs are conducted independently of, or in conjunction with, environmental impact assessments (3,40). Further work could clarify the best means with which to incorporate health issues into environmental impact assessment processes.

HIAs can directly influence decisions outside the health sector and frequently lead to other benefits that may have long-term health promotion value for communities. An aggregation of factors is necessary for HIA success. A central objective of HIAs is to “have health at the table” when decisions are being made; the impact evaluation reports reviewed suggest that this purpose generally is being fulfilled.
